# Orthodontia, Its Significance to the General Dentist

**Published:** 1908-09-15

**Authors:** James D. Locke


### ORTHODONTIA, ITS SIGNIFICANCE TO THE GENERAL DENTIST.

BY JAMES D. LOCKE, D.D.S.

Read before the Michigan Fifth District Society, June 8, 1908.

In our elementary algebra problems we were told to let X represent so many marbles in Johnny's right hand. In all mathematics we have an X or working basis from which we evolve our most intricate problems.

In Orthodontia, which only within the past few years could be termed a true science, we again make use of the time-honored X in the form of the Permanent First Sup Molar, which is popularly designated, the key to occlusion, and by application of the working knowledge which has been given to us in regard to this tooth we readily determine whether or not the dental arches are normal in their relation one to the other.

Previous to this the question of diagnosis in cases of malocclusion, resolved itself into little more or less than mere guess work, each man using his best judgment, without any fixed rule to guide him as to the form of abnormality which presented itself for correction, and pursued the mode of treatment which to him seemed advisable, and we all know some of us, perhaps from personal experience, how uncertain were the results, perchance an improvement, but more often an irreparable damage.

After many years of groping in the dark and not being satisfied with the results of his predecessors, Dr. Angle, finally presented this to the dental profession; so simple, but as yet undiscovered, or if it had been discovered it was not an accepted scientific fact, that the first permanent sup molar at all times occupied a positive position in the sup max bone in its relation to the skull, mesio-distally.

Bucco-lingually, this tooth, from abnormal functions of the tongue or muscles of the cheek, may be deflected from its normal position, but mesio-distally never.

Let us study some of the causes which work to bring about this permanency of position of this tooth.

We all know that the first permanent molar erupts at the sixth year of child life when all of the deciduous set are supposed to be in position with the exception of the four perm incisors, which may be erupting simultaneously, and all the dentists present who were so fortunate as to have seen Dr. Runyan's interesting slides at the recent dental meeting held here will no doubt remember having seen the slide which represented the child set at six years, showing the permanent first molars erupted and the crowns of the second molars and bicuspids formed and the roots developing, practically locking and holding the first molars in an immovable position in the jaw mesio-distally.

Any lengthening or developing in the maxillary bones after the eruption of this tooth must take place anterior to and posterior to this member.

So much for the permanency of position of the first molar mesio-distally, which point I hope to have imprinted indelibly upon your mind. Let us go back for a few brief moments and consider it merely at X or something problematical from which we are going to make a deduction; and, gentlemen, the question of classification in all cases of maloccusion with X, or our First Sup Molar as the key from which we are working, is nothing more or less than a mathematical deduction, which any ordinarily bright child old enough to erupt its second molars, could readily be made to understand, as easily as they could be taught to do a problem in school, so clearly and exactly has this information been put before us.

There is nothing that prohibits a dentist in general practice from receiving a knowledge of these different forms of abnormalities, and each man should make himself thoroughly conversant with them, that he might be able to diagnose these conditions in the little patients brought to him, and so talk intelligently to the child's parents, explaining the evil effects that might accrue were the abnormal conditions not corrected, and so enhance his professional services to his clientele and at the same time pave the way for the orthodontist.

The benefit is two-fold, as a few reassuring words in advance from the family dentist is equivalent to many times the attempts on the part of the orthodontist, to convince the parent that such and such is the case.

Yet what is the significance of these different classes of malocclusion and their accompanying evils to the busy practicing dentist, whose time and thought are taken up with the many other branches of our profession, unless he has given it some study during his leisure hours?

Yet a few brief words dealing comprehensively with the fundamental principles of this subject, would furnish the working which would enable him to properly diagnose these cases, making him an ally of the orthodontist, examining the mouths of the little patients brought to him by the fond parents for his best advice, noting if the occlusion is normal or abnormal, and if nature is performing her full duty and there is taking place a harmonious development of the maxillary bones, shedding of the baby set, and erupting of the permanent teeth. Instead of receiving this elementary knowledge he has thrust upon him, something of a technical nature in which, no matter how much he may be interested at the time, it is not applicable in his every day practice, and he soon forgets, for he has learned to speak and read before he has learned his alphabet,

Tonight I hope to give something of the A, B, C of Orthodontia, if within my power sufficiently to interest you in this subject, that you may learn to distinguish between these different classes of malocclusion and some of the causes attendant thereon.

Let us now take up Class I, which, according to Dr. Angle, embraces about 70 per cent of all malocclusion.

The chief characteristic of this class is the normal relation of the dental arches, mesio-distally, which is denoted by the bucco-mesial cusp of the First Sup Molar striking into the groove formed midway between the bucco-mesial and bucco-distal cusps of the Lower First Molar, which is the simple, yet efficient locking device which nature has provided for retaining the lower jaw in its normal relation to the upper.

In this class of cases we have our abnormality confined to the front of the mouth, and takes the form of a bunching of the teeth, the arches being more or less reduced in size as a result.

The causes of the irregularity in this class are treated under two heads—constitutional and acquired. Under the head of constitutional we find a very common cause is a lack of development in the inferior maxillary bone in the area extending between the deciduous cuspid teeth, causing some or all of the lower incisors on eruption to be deflected from their normal course.

The abnormality here, through occlusion, denotes itself in the opposite arch by carrying the antagonizing teeth out of line. Lack of anterior occlusion, or what is familiarly known as open bite, is where we find no occlusion until we reach the First or Second Molars, is attributed to a lack of development in the ramus of jaw or excessive development in posterior portion of body of bone.

Lack of development in the inter-maxillary bones sometimes produces irregularity in the anterior part of the upper arch.

Under the head of acqnired, we find the pernicious habits that children form in early life constitute many of the causes of irregularity, such as abnormal tongue or lip habits. thumb sucking, etc., and at this time I should like to impress you with the importance of watching your little patients for any of these bad habits and enlist the parents' co-operation in an endeavor to correct the child of these abnormal functions and thus early prevent these abnormalities with their resultant embarrassment.

The question of too early or tardy extraction of deciduous teeth is one which is of vital importance to the proper eruption of the permanent teeth and one which requires the dentist's best judgment.

You must study the little jaws to see if they are developing properly and note if there is any sign of the permanent tooth in the alveolus; and if so, and if the root of the deciduous tooth is not being absorbed, and after the proper time it shows no sign of loosening, extract it. But be careful of the deciduous cuspid that you may not extract it too soon and so allow the space to close between the lateral incisor and the first bicuspid, causing the permanent cuspid to be deflected from its normal course and erupt out of line. A simple appliance adjusted in many cases where deciduous teeth have been lost prematurely will retain the space and allow the permanent tooth to wedge itself into its proper position in the arch.

The peculiar shape of the deciduous molars in that their crowns occupy a much greater space mesio-distally in the arch than their roots necessitates a full contouring of fillings inserted in their proximate surfaces, as these operations become a great factor in either regaining or losing a considerable amount of the normal space which nature has provided in the arch for the proper eruption of the bicuspids.

Death of a pulp in a deciduous tooth will prevent its proper physiological resorption and cause it to be removed by a pathological process. Extraction in these cases, many times prove essential.

The extraction of permanent teeth and the attendant evils is a topic with which all of the dentists present are too familiar for me to dwell on, yet there is one phase of the question I wish to touch, and that is, by all means avoid extracting to relieve a crowded arch, unless it be a supernumary tooth, because in your endeavor to alleviate the irregularity you are robbing the face of just so much of its normal contour.

Class II, with its subdivisions, represents that condition familiarly known among the laity as receding chin. It embraces that type in which the lower jaw is in distal occlusion with the upper in one or both of its lateral halves, or the lower first molar on one or both sides is occluding posterior to the upper first molar. In this class the cause of this morbid condition is entirely constitutional. The air passages being partially or almost entirely closed, from adenoid growths in the naso-pharnx, deflected septum, hypertrophied turbinated bones, polypi or enlarged tonsils, the child in its endeavor to establish a new breathing tract, is forced to keep the mouth open, more easily admitting the flow of air to and from the lungs.

The evil effects here denote themselves in the occlusion at the time of the eruption of the six-year molars. The cusps of the deciduous teeth being worn from mastication, and the depressor muscles drawing the jaw downward and backward, thwarts nature in its endeavor to lock the occlusion in the permanent set by the proper indigitation of the cusps of the six year molars.

These are cases which give the dentist the greatest opportunity of best serving his little patients by watching for these conditions, and if he has any suspicion of mouth-breathing have the child's throat and nose examined for these foreign growths by a competent Rhinologist, and so save the child considerable inconvenience in after life.

A slight operation, if performed early, will prevent considerable orthodontia and wonderfully improves the child's health and faculties, to say nothing of the facial deformity you are preventing. Children are now regularly examined in many public schools for these conditions, but in a great many cases the child has reached an age where the abnormality has already denoted itself in the occlusion. If dentists would but give this question the proper thought, they would soon find themselves becoming greatly interested. and would learn from facial characteristics in a large percentage of cases to diagnose this condition in a child at sight.

Class III, with its subdivisions, represents that type of cases commonly known among the laity as undershot, or protruding chin. In this class we have the lower jaw in one or both of its lateral halves in mesial occlusion with its upper, or the lower first molar on one or both sides occluding anterior to its opposing member, the upper first molar.

The causes and accompanying evil here are practically the same as in Class II, except in a type of cases where we find an inherent tendency to over-development in the ramus or in the body of the inferior maxillary bones in the area occupied by the bicuspid teeth.

A peculiarity in this class of malocclusion is that the teeth in each arch may not early present any great irregularity, and thus the condition escapes the notice of the dentist unless he is watching the occlusion of his young patients, until the abnormality shall have become so intensified and the ramus of the jaw shall have become so ossified in its abnormal form that it is too late for the Orthodontist to approach an ideal result.

In conclusion, there is one vital point I should like to leave fresh in your memory, and that is, by all means do not belie the confidence your patients put in your professional judgment by advising parents to await the eruption of the entire permanent dentition that nature may assert herself in an endeavor to correct irregularities.

In proportion to the length of time the operation is delayed, so will the abnormality intensify.

When a tooth is deflected from its normal course, there is always a primary reason, and once it has assumed a malposition it not only carries other teeth in its own arch out of their proper positions, but through perverted occlusion will destroy the alignment in the opposite arch, and lastly bear this point in mind and carry it into your practice, that it is easier to guide a tooth into its proper position in the arch than it is to make space and mechanically draw that tooth into the arch with many agonizing forces combating you.

